# Hydroxychloroquine: a comprehensive review and its controversial role in coronavirus disease 2019

**DOI:** 10.1080/07853890.2020.1839959

**Published:** 2020-11-02

**Authors:** Pankaj Bansal, Amandeep Goyal, Austin Cusick, Shubham Lahan, Harpal S. Dhaliwal, Poonam Bhyan, Pradnya Brijmohan Bhattad, Fawad Aslam, Sagar Ranka, Tarun Dalia, Lovely Chhabra, Devang Sanghavi, Bhavin Sonani, John M. Davis

**Affiliations:** a Mayo Clinic Health System, Eau Claire, WI, USA; b University of Kansas Medical Center, Kansas City, KS, USA; c Ohio University Heritage College of Osteopathic Medicine, Athens, OH, USA; d University College of Medical Sciences, New Delhi, India; e Christian Medical College and Hospital, Ludhiana, India; f Cape Fear Valley Hospital, Fayetteville, NC, USA; g East Tennessee State University, Johnson City, TN, USA; h Mayo Clinic, Scottsdale, AZ, USA, USA; i Heartland Regional Medical Center, Southern IL University, Carbondale, IL, USA; j Mayo Clinic, Jacksonville, FL, USA; k HSHS St John’s Hospital, Springfield, IL, USA; l Mayo Clinic, Rochester, MN, USA

**Keywords:** Hydroxychloroquine, COVID-19, cardiotoxicity, mechanism of action

## Abstract

Hydroxychloroquine, initially used as an antimalarial, is used as an immunomodulatory and anti-inflammatory agent for the management of autoimmune and rheumatic diseases such as systemic lupus erythematosus. Lately, there has been interest in its potential efficacy against severe acute respiratory syndrome coronavirus 2, with several speculated mechanisms. The purpose of this review is to elaborate on the mechanisms surrounding hydroxychloroquine. The review is an in-depth analysis of the antimalarial, immunomodulatory, and antiviral mechanisms of hydroxychloroquine, with detailed and novel pictorial explanations. The mechanisms of hydroxychloroquine are related to potential cardiotoxic manifestations and demonstrate potential adverse effects when used for coronavirus disease 2019 (COVID-19). Finally, current literature associated with hydroxychloroquine and COVID-19 has been analyzed to interrelate the mechanisms, adverse effects, and use of hydroxychloroquine in the current pandemic. Currently, there is insufficient evidence about the efficacy and safety of hydroxychloroquine in COVID-19.

**KEY MESSAGES**
1. HCQ, initially an antimalarial agent, is used as an immunomodulatory agent for managing several autoimmune diseases, for which its efficacy is linked to inhibiting lysosomal antigen processing, MHC-II antigen presentation, and TLR functions.
2. HCQ is generally well-tolerated although severe life-threatening adverse effects including cardiomyopathy and conduction defects have been reported.
3. HCQ use in COVID-19 should be discouraged outside clinical trials under strict medical supervision.

## Introduction

Initially used to treat malaria, hydroxychloroquine (HCQ) is an important therapeutic option for several autoimmune diseases, especially systemic lupus erythematosus (SLE) and rheumatoid arthritis (RA). The efficacy of HCQ in rheumatic illnesses stems from its anti-inflammatory and immunomodulatory effects, the mechanisms of which are unclear. Although initially thought to exert its immunomodulatory effects by interfering with lysosomal enzymatic actions and major histocompatibility complex class-II (MHC-II)-mediated antigen presentation, emerging evidence suggests interference with Toll-like receptor (TLR) functions as an additional pathway [1].

HCQ is one of the safest immunomodulatory agents for rheumatic illness. However, rare but serious adverse effects have been reported, mostly with long-term use. HCQ-induced acquired lysosomal storage disease causes some of these adverse effects, including myopathy and cardiomyopathy [1]. Corrected QT (QTc) interval prolongation is associated with HCQ owing to human ether-à-go-go-related gene (hERG) voltage-gated potassium channel inhibition [2].

*In vitro* studies [3–7] have revealed the antiviral properties of HCQ, raising interest in its potential therapeutic role in coronavirus disease 2019 (COVID-19). As of 30 August 2020, 24,854,140 cases of COVID-19 were reported with 838,924 deaths globally according to the World Health Organization (WHO) [8] and currently there is no effective treatment for this novel disease. Although HCQ was among the first drugs evaluated for COVID-19 treatment, clinical trials [9] reported so far have largely been inadequate to confirm its efficacy owing to poor methodology and small sample sizes. Furthermore, recent studies [10–15] have raised concerns about the safety of HCQ, especially in combination with other drugs.

This review illustrates the mechanisms of action underlying the antimalarial, immunomodulatory, and potentially antiviral properties of HCQ, and the pathophysiological aspects of HCQ-mediated cardiotoxicity, with novel pictorial explanations. Additionally, the controversial role of HCQ for COVID-19 treatment is summarized with currently available clinical trials.

## Search strategy and selection criteria

Data for this review were identified by searches of MEDLINE, PubMed, EMBASE, SCOPUS, Google Scholar, Science Citation Index and references from relevant articles using the search terms “hydroxychloroquine,” “severe acute respiratory syndrome coronavirus 2,” “SARS-CoV-2,” “COVID-19,” “2019-nCoV,” “Wuhan,” and “coronavirus.” Only articles published in English from inception to 31st August 2020, restricted to humans, and directly related to this review were included.

## Indications of HCQ

Antimalarials have been used for the treatment of RA since the 1950s. HCQ is one of the mildest and safestdisease-modifying antirheumatic drugs [16]. Although initially used predominantly for RA, HCQ may be most efficacious in SLE, for which besides treating skin and joint disease, HCQ prevents disease flares, promotes long-term survival, and improves overall prognosis [17]. Furthermore, the antithrombotic effects of HCQ are beneficial in patients with SLE and anti-phospholipid syndrome [18,19]. HCQ may have utility in several infectious disease processes. Although the overall efficacy of HCQ in infectious diseases, besides malaria, is unknown, HCQ is being explored in human immunodeficiency viruses, *Coxiella burnetii*, Zika virus, chikungunya, and Whipple’s disease [18].

## Mechanisms of action

HCQ and chloroquine (CQ) are 4-aminoquinolines with similar chemical structures, except for an ethyl group substitution by a hydroxyethyl group on the tertiary amino acid side chain in HCQ. HCQ, 2-[[4-[(7-chloro-4-quinolyl)amino]pentyl]ethylamino]ethanol sulphate [20], has antimalarial and immunomodulatory properties. HCQ is absorbed rapidly after oral administration and has a long half-life of 30–60 days, reaching steady plasma levels up to 6 months after therapy initiation [21]. HCQ can be detected in plasma and tissues several months to years after discontinuation [22]. HCQ is metabolized in the liver by CYP450 and undergoes renal excretion.

### Antimalarial action

HCQ was widely used as an antimalarial agent before the rapid development of drug-resistance. In malaria, HCQ acts as a blood schizonticide against trophozoites in red blood cells (RBCs). In RBCs, a trophozoite obtains the amino acids required for growth by haemoglobin breakdown in its food vacuole. A by-product of this breakdown is haem (ferriprotoporphyrin IX), which is toxic to the parasite as it lyses cell membranes. In the food vacuole, this toxic haem is converted to non-toxic crystallized hemozoin [23].

**Figure 1.**
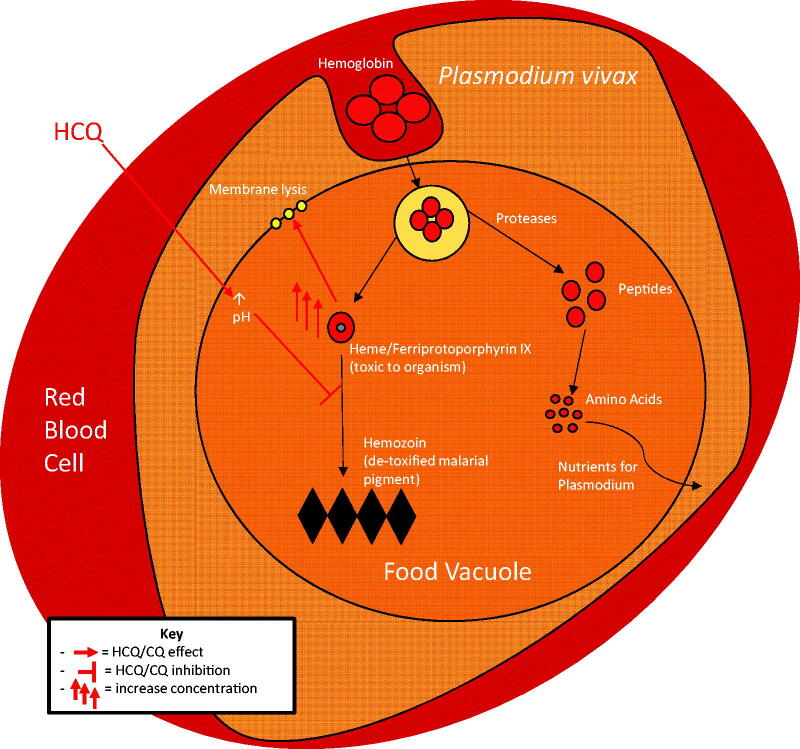
Antimalarial actions of hydroxychloroquine (HCQ). Being lipophilic, HCQ easily permeates the red blood cell that contains the malaria parasite and enters the food vacuole of the parasite. Being weakly alkaline, HCQ increases the pH of the food vacuole, which inhibits the conversion of toxic haem to non-toxic hemozoin. Accumulation of toxic haem leads to membrane lysis and parasite death.

The antimalarial action of HCQ is dependent on its lipophilicity to permeate and accumulate in intracellular structures, including lysosomes and food vacuoles of the malaria parasite. Once inside an intracellular vesicle, HCQ increases the pH as a weak base [24]. By increasing food vacuole pH, HCQ interferes with the conversion of haem to hemozoin, thereby increasing the toxic haem level, which lyses the parasite (Figure 1) [18].

### Immunomodulatory and anti-inflammatory action

Although HCQ is efficacious in several autoimmune and inflammatory disorders, including SLE and RA, the exact mechanism underlying the anti-inflammatory and immunomodulatory actions of HCQ is unclear.

**Figure 2.**
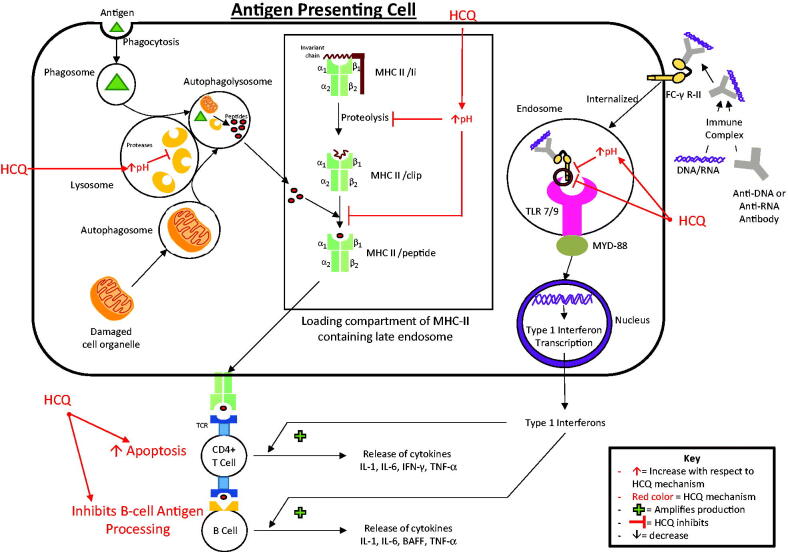
Immunomodulatory actions of hydroxychloroquine (HCQ). In antigen-presenting cells, HCQ increases the pH of lysosomes and inhibits lysosomal proteases, thereby inhibiting antigen processing and presentation to major histocompatibility complex class-II proteins (MHC-II). HCQ increases the pH of the late endosome loading compartment that contains MHC-II, which inhibits the clipping and replacement of the invariant chain (Ii) by antigenic peptides and prevents the formation of the MHC-II/peptide complex, thereby inhibiting MHC-II-mediated antigen presentation to CD4+ T-cells. In plasmacytoid dendritic cells, HCQ inhibits immune complex-mediated Toll-like receptor (TLR) 7 and 9 in the endosome by increasing the pH of the endosome and directly inhibiting the binding of the immune complex to the TLR 7 and 9, thereby preventing downstream type-1 interferon transcription. HCQ promotes T-cell apoptosis and inhibits B-cell antigen processing, thereby decreasing T-cell- and B-cell-mediated cytokine release.

Being lipophilic, HCQ easily permeates cell membranes and accumulates in intracellular vesicles, including lysosomes, endosomes, and autophagosomes. In these acidic vesicles, it interferes with vesicular enzyme functionality (such as proteases) by increasing the pH [25]. In antigen-presenting cells (APCs), HCQ interferes with the processing of antigens to peptides, thereby preventing peptide presentation for MHC-II [1,26]. Furthermore, in the loading compartment of MHC-II-containing acidic endosomes, HCQ possibly interferes with the interaction of peptides with MHC-II. A crucial step in this interaction is the clipping of the MHC-II invariant chain and replacement by antigen peptides, which forms the MHC-II/peptide complex. An increase in pH caused by HCQ inhibits invariant chain clipping by proteases. This selectively inhibits the binding of low-affinity self-antigen peptides to the MHC-II binding site but not of high-affinity foreign-antigen peptides (such as bacterial peptides), possibly explaining why HCQ is not associated with an increased infection risk [1,21] (Figure 2).

Thus, by interfering with MHC-II-related autoantigen presentation to cluster of differentiation (CD) 4+ T-cells *via* APCs, HCQ interferes with cytokine release. This action also interferes with B-cell activation by CD4+ T-cells. Additionally, HCQ induces apoptosis of autoreactive T-cells and interferes with antigen processing by B-cells, thereby interfering with their functions and cytokine production (interleukin [IL]-1, IL-6, interferon-gamma, tumour necrosis factor [TNF], and B-cell activating factor) [21,27].

A recently highlighted immunomodulatory mechanism associated with HCQ is the inhibition of TLR signalling pathways [28]. Immune complexes that contain DNA or RNA bind Fc-gamma receptor-II on plasmacytoid dendritic cells and are internalized to endosomes that contain intracellular TLR7 and TLR9, which recognizes single-stranded RNA and DNA respectively. The binding of immune complexes to TLR7 and TLR9 leads to the downstream induction of type-1 interferon transcription through the myeloid differentiation primary response protein 88. The pathogenic role of type-1 interferons in various rheumatic diseases, such as SLE, has been well described [29]. Type-1 interferons activate T-cells, B-cells, natural killer cells, myeloid dendritic cells, and monocytes, leading to further cytokine production [1,21]. HCQ accumulates in TLR7 and TLR9-containing endosomes and directly inhibits the binding of TLR7 and TLR9 to the immune complexes. By increasing the pH of the endosome, HCQ can also interfere with TLR processing [30]. Thus, by interfering with TLR7 and TLR9 signalling, HCQ inhibits the transcription of type-1 interferons, which results in immunomodulatory and anti-inflammatory effects [1,21] (Figure 2).

### Antiviral action

**Figure 3.**
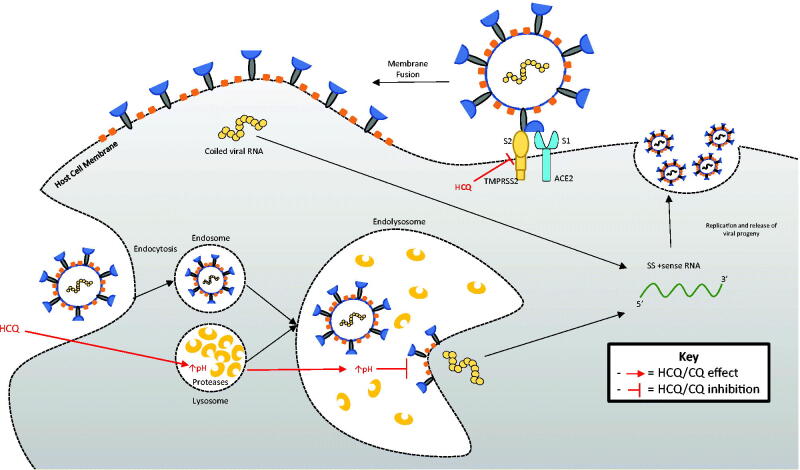
Proposed theoretical antiviral actions of hydroxychloroquine (HCQ). By increasing the pH of the lysosome, HCQ may inhibit endosomal acidification to prevent viral RNA shedding into the cytoplasm, thereby interfering with downstream viral replication. HCQ may bind the gangliosides and inhibit the communication between the spike protein and the cell membrane, thus inhibiting viral entry into the cell.

Owing to the current COVID-19 pandemic, several therapies are under investigation for potential efficacy against severe acute respiratory syndrome coronavirus 2 (SARS-CoV-2) using current and historical data. *In vitro* studies have shown potential antiviral properties associated with HCQ and CQ, raising interest in their role as potential therapies against SARS-CoV-2. The anti-inflammatory action of HCQ is dependent on immunomodulation and the downstream production of cytokines. The attenuation of inflammation results in a successful response in a rheumatic setting and possibly SARS-CoV-2 infection [31]. Furthermore, successful SARS-CoV-2 entry into host cells is strongly dependent on angiotensin-converting enzyme-2 (ACE-2) interaction with the viral spike protein [32]. CQ reduces the glycosylation of ACE-2, which inhibits the binding of the SARS-CoV-2 spike protein to the cell surface and cell integration [33,34]. Recent investigation also suggests that by binding the gangliosides, HCQ inhibits communication between the spike protein and the cell membrane, thus inhibiting viral entry into the cell [33]. Additionally, HCQ and CQ accumulate in lysosomes and, by increasing the pH of lysosomes, prevent viral particle release by disrupting vital cellular pathways [34]. Moreover, the inhibition of glycosyl-transferases, post-translational viral modification, quinone reductase-2 and sialic acid synthesis, and viral replicative mechanisms is implicated in the antiviral effect of HCQ (Figure 3) [31]. However, to date, no *in vivo* studies have confirmed the potential antiviral action of HCQ in humans.

## Adverse effects

HCQ is widely used in rheumatology and is generally safe and well-tolerated; however, several adverse effects have been reported, some irreversible and life-threatening [35].

### Cardiotoxicity

**Figure 4.**
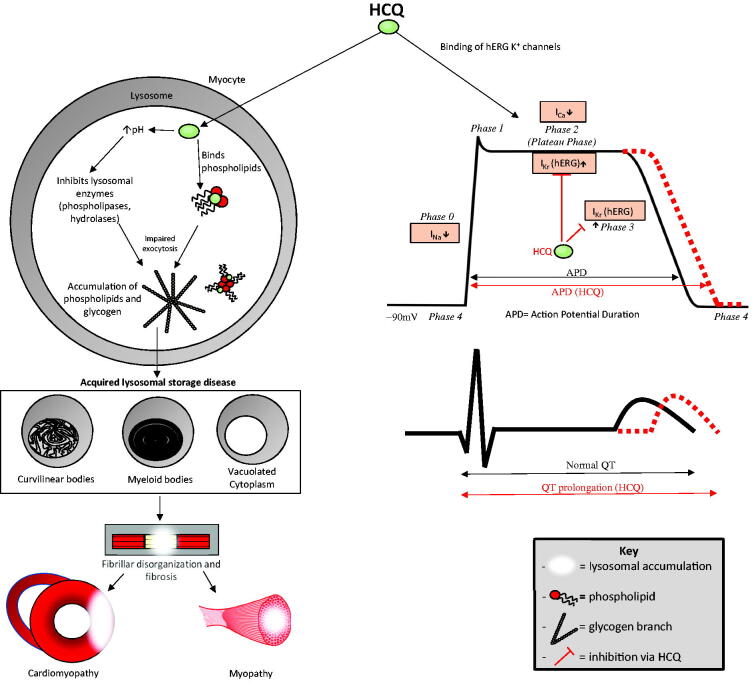
Hydroxychloroquine (HCQ)-induced cardiomyopathy and myopathy. HCQ permeates the lysosomes of myocytes and causes glycogen and phospholipid accumulation by binding phospholipids and increasing the pH, thereby inhibiting phospholipases and hydrolases. This leads to the formation of curvilinear and myeloid bodies and cytoplasmic vacuoles causing an acquired lysosomal storage disease, which causes fibrillar disorganization, atrophy, and fibrosis. These changes lead to cardiomyopathy and proximal myopathy in skeletal muscles. HCQ-induced conduction abnormalities. HCQ binds I_kr_ (hERG) potassium channels, slowing potassium efflux in phase 2 and especially phase 3, thereby prolonging the action potential duration that leads to QTc prolongation (depicted in red). The action potential begins with sodium influx, phase 0; rapid potassium efflux, phase 1; calcium influx balanced by potassium efflux, phase 2; potassium efflux, phase 3; and subsequent restoration of resting membrane potential, phase 4.

Cardiomyopathy and conduction abnormalities with HCQ have been described and recently highlighted with its use for COVID-19. Unlike cardiomyopathy, which is rare and occurs after prolonged exposure to HCQ, conduction abnormalities are common and acute (Figure 4) [36].

Acquired lysosomal storage disease induced by HCQ is the pathogenetic pathway for the development of cardiomyopathy, mostly with long-term use [37]. Most cases are caused by accumulation, which can be augmented by CYP450 2C8 mutation [38]. Being lipophilic, HCQ easily permeates myocytes, in which it binds lysosomal phospholipids, leading to lysosomal accumulation of phospholipids. Furthermore, by increasing the pH of the lysosome, HCQ inhibits lysosomal enzymes, such as hydrolases and phospholipases, which interferes with lysosomal function and exocytosis, leading to the accumulation of glycogen and phospholipids [39]. The abnormal accumulation of metabolic products and lysosomal inclusions in cardiac myocytes induces an acquired lysosomal storage disease, leading to myofibrillar disorganization, atrophy, and fibrosis, which may lead to cardiomyopathy [40]. Acquired lysosomal storage disease can be visualized by electron microscopy as vacuoles, myeloid bodies, and curvilinear bodies. Although vacuoles are more commonly detected, curvilinear bodies are pathognomonic of HCQ-induced lysosomal storage disease. Histopathologically, HCQ-induced lysosomal storage disease appears identical to inherited lysosomal storage diseases, including Anderson-Fabry disease, except for the presence of curvilinear bodies [41]. The most frequent clinical presentation is acute exacerbation of right, left, or biventricular heart failure. Risk factors for the development of HCQ-induced cardiomyopathy include prolonged exposure to the drug (several years), elderly age, renal insufficiency, and chronic liver disease. Diffusely thickened ventricular walls on a transthoracic echocardiogram are hallmarks of this cardiomyopathy, although this is not specific to HCQ-induced cardiomyopathy [42,43]. A cardiac magnetic resonance image (MRI) that shows late gadolinium enhancement is a marker for fibrosis, especially in hypertrophic cardiomyopathy, and prognostic marker for cardiac death. MRI can also be used to guide biopsy sampling; hence, MRI plays an important role in cardiac evaluation and risk stratification [44]. Endomyocardial biopsy with electron microscopy is the most specific diagnostic test [45]. Fortunately, most patients report symptom resolution after drug cessation [46].

HCQ-induced conduction disorders are usually acute, owing to cardiac channel blockage. Several structurally-related medications, such as quinolones, CQ, and HCQ, that affect myocardial depolarization and repolarization mainly *via* cardiac K^+^ channel blockage cause QT/QTc prolongation, which is an indicator of an increased risk of drug-induced torsade de pointes (TdP). TdP is usually self-limiting but can degenerate into lethal ventricular fibrillation and cause sudden cardiac death [38]. The main mechanism of HCQ-induced QT prolongation is blockage of hERG K^+^ channels [47]. *hERG*, located on chromosome 7 q35-36, encodes the pore-forming subunits of hERG K^+^ channels, which mediate rapid delayed rectifier potassium currents (Ikr), resulting in phase 2 and phase 3 of repolarization in the cardiac cycle. The blockage of hERG K^+^ Ikr channels increases the duration of phase 2 and especially phase 3 repolarization, leading to a prolonged QT interval (class III antiarrhythmic effect) [2]. Additionally, HCQ can cause hypotension owing to alpha blockade, leading to arteriolar and venular dilation, sodium (class 1 antiarrhythmic effect) and calcium channel blockage, and a negative inotropic effect at low micromolar concentrations [2]. These effects explain the reduction in the maximum velocity of cardiac action potential and conduction disturbances, such as atrioventricular block, bundle branch block, and a QT prolongation effect [38]. HCQ blocks cardiac channels in a dose-dependent manner. At the currently recommended dose of less than 5 mg/kg/day, HCQ is usually safe, although prolongation of the QT/QRS is rarely observed on a surface electrocardiogram [48]. Ventricular ectopy and lethal ventricular arrhythmias have been reported, mostly with supra-therapeutic doses. Risk factors for the development of lethal ventricular arrhythmias include underlying structural heart disease, electrolyte abnormalities (e.g. hypokalaemia and hypomagnesemia), female sex, elderly age, genetic defects of cardiac ion channels (inherited long QT syndrome owing to *hERG* mutation), renal insufficiency, chronic liver disease, and, concomitant use of other drug classes that cause QT prolongation, such as azithromycin (AZ) [2]. A QT/QTc interval of over 500 ms is associated with a higher risk of TdP and sudden cardiac death. The risk of cardiotoxicity secondary to HCQ is theoretically greater in critically ill patients with COVID-19 owing to the potential for viral myocarditis, cardiac injury owing to cytokine storm, and multiorgan failure [47].

### Other adverse effects

HCQ-induced ocular toxicity has been recognized, especially Bilateral bull’s eye maculopathy with central macular involvement sparing the parafovea, which is observed rarely (< 1%) in the first 5 years of therapy, in < 2% of cases after 10 years, and in up to 20% of cases after 20 years [49,50]. HCQ binds melanin in the retinal pigment epithelium (RPE) and accumulation results in macular damage. Furthermore, by increasing RPE lysosome pH and inhibiting lysosomal enzymes and phagocytosis, HCQ inhibits the clearance of shed outer photoreceptor segments, leading to accumulation. This leads to the migration of pigment-containing RPE cells to outer retinal layers, and in the loss of photoreceptors and RPE atrophy. Damage to the outer retinal photoreceptor layer precedes RPE damage and atrophy according to optical coherence tomography data. HCQ-induced retinopathy is irreversible and may continue for several months after drug discontinuation owing to its long half-life [49,51]. HCQ can also bind cellular lipids in the cornea and deposit in the corneal basal epithelial layer leading to corneal deposition (vortex keratopathy), which is reversible after drug discontinuation. Other ocular adverse effects include ciliary body deposition leading to disturbances in accommodation and blurred vision, which are also reversible [52].

Proximal myopathy, possibly associated with neuropathy, owing to HCQ has been reported, with a similar pathogenesis to that of HCQ-induced cardiomyopathy, i.e. acquired lysosomal storage disease (Figure 4). Risk factors include higher cumulative dose, elderly age, and renal disease. HCQ-induced myopathy presents with proximal weakness with normal creatine phosphokinase levels but abnormal electromyogram and muscle biopsy results that reveal vacuoles, myeloid bodies, or curvilinear bodies, the latter being the most specific to this disease [53]. HCQ-induced myopathy is usually reversible, with rapid clinical improvement after drug discontinuation [54].

Other adverse effects of HCQ include gastrointestinal distress (e.g. nausea, vomiting, diarrhoea, abdominal pain, and anorexia) and skin rash, which are common and observed in 5–10% of patients administered with HCQ [16,55]. Rare adverse effects include skin hyperpigmentation, alopecia, agranulocytosis, aplastic anaemia, leukopoenia, thrombocytopenia, haemolytic anaemia in glucose-6-phosphate dehydrogenase deficiency, irritability, nervousness, headaches, dizziness, vertigo, tinnitus, and transaminitis [56].

## COVID-19 and HCQ literature review

In December 2019, China reported a novel viral illness caused by SARS-CoV-2, later defined by the WHO as COVID-19. HCQ gained interest as a potential therapeutic option for COVID-19 based on *in vitro* studies suggesting efficacy of HCQ and CQ against SARS-COv and SARS-Cov-2 [3–7] (Table 1). Although the initial studies showed potential efficacy, these had several flaws and the risk of bias and several further trials failed to confirm the efficacy of HCQ for COVID-19.

**Table 1.** Summary of *in vitro* studies with hydroxychloroquine and chloroquine in coronaviruses.

Date	Authors	Results
August 2004	Keyaerts et al. [3]	CQ inhibits SARS-CoV
August 2005	Vincent et al. [4]	CQ inhibits SARS-CoV
February 2020	Wang et al. [5]	CQ inhibits SARS-CoV-2
March 2020	Yao et al. [6]	HCQ > CQ against SARS-CoV-2, dosage recommendations
April 2020	Andreani et al. [7]	HCQ and AZ show synergistic effect against SARS-CoV-2

### Studies suggesting HCQ efficacy in COVID-19

HCQ efficacy investigation began with a French investigation of 36 patients [57]. The investigation claimed efficacy of HCQ ± AZ in COVID-19 as significantly more patients administered HCQ ± AZ had negative polymerase chain reaction (PCR) results on Day 6 than those not administered HCQ. However, this study had several major limitations, including small sample size, lack of randomization and blinding, heterogeneous patient recruitment, and poorly selected endpoints. Furthermore, the six patients lost to follow-up were all in the treatment arm, some with adverse outcomes. Despite several significant concerns with the methodology, this study gained widespread attention that led to the use of HCQ in patients with COVID-19. Another study by the same authors evaluated the efficacy of HCQ with AZ in 80 patients with COVID-19 and showed efficacy in 65 patients [58]. However, a significant pitfall of this study was the lack of a control arm. Furthermore, the viral PCR threshold value, which determined patient discharge from the hospital, was changed multiple times during the study.

The first randomized controlled trial (RCT) suggesting efficacy of HCQ in COVID-19 was reported by Chen et al., who reported a shorter duration of symptoms (fever and cough) and radiographic improvements in patients with mild COVID-19 treated with HCQ for 5 days compared with those with standard of care treatments [59]. However, this study was also limited by sample size (31 patients in each arm), strict inclusion criteria excluding severe cases, which raises concerns of selection bias. Additionally, clinical improvement was assessed by only fever and cough, excluding other important outcomes, such as oxygen saturation. Another open-label RCT by Chen et al. suggested shorter time to clinical recovery which was 5.50 days in CQ arm (*n* = 18), 6.00 days in HCQ arm (*n* = 18) and 7.50 days in control arm (*n* = 12). Besides the small sample size, this study was nonblinded. Further, the study was terminated early and was underpowered [60].

The recent evidence about the efficacy of HCQ in COVID-19 has been through retrospective case series or retrospective non-randomized, non-blinded observational studies. Ahmad et al. reported clinical recovery defined as improvement in fever and dyspnoea in 85% of the patients hospitalized with COVID-19 when treated with HCQ and doxycycline. This case series had a small sample size (*n* = 54) with no control arm. Further, 14.8% (8) patients in this study clinically deteriorated or died and radiographic improvement was observed only in 11% of the patients [61]. Million et al. reported “good clinical outcome” and virological clearance in 91·7% out of 1,061 patients with COVID-19 treated with HCQ + AZ [62]. However, drawbacks of this retrospective case series included no control arm, poorly defined clinical outcomes, unsupervised treatment, and incomplete data with computed tomography scans and serum drug levels unavailable in some cases. Yu et al. reported decreased mortality (18·88% vs 45·8%) and reduced IL-6 levels with the use of HCQ in 48 critically ill patients with COVID-19 [63]. Antivirals were used in several patients and significantly more patients receiving interferon and antibiotics in the non-HCQ group than in the HCQ group. Additionally, with the cause of mortality not specified, drug interactions and comorbidities could have influenced the results. Novales et al. reported decreased mortality in patients treated with HCQ (27 out of 123, 22%) compared to the control arm (21 out of 43, 48.8%) in a retrospective analysis in patients admitted with COVID-19. Besides small sample size, lack of blinding and retrospective study method, other limitations of this study included use of other antiviral and anti-inflammatory medications, younger patients in the HCQ arm (61.5 years vs 68.7 years), and cause of mortality not specified [64]. A large retrospective population wide analysis of patients with confirmed/suspected COVID-19 from Portugal compared the incidence of PCR positivity in those who were already on HCQ to those who were not [65]. About, 0.29% of all patients who tested positive for COVID-19 were on HCQ while 0.36% of all patients who tested negative were on HCQ. No data was available about patient comorbidities, and drug compliance in this retrospective analysis. A direct causation effect could be deduced based on this retrospective observational analysis [65]. A recent multicenter retrospective analysis of HCQ ± AZ in 2,541 inpatients with COVID-19 reported significantly lower mortality in patients treated with HCQ alone (13.5%), or in combination with AZ (20.1%) than in patients in the control arm (26.4%) [66]. However, significantly more patients received corticosteroids (78·9% and 74·3% versus 35·7%) and tocilizumab (3·4% and 9·2% versus 1·2%) in the HCQ and HCQ + AZ arms than in the control arm. Furthermore, significantly more patients were more than 65 years old (61·4% versus 48·9% and 45·5%) in the control arm than in the HCQ and HCQ + AZ arms. Although propensity score matching suggested a 51% decline in the mortality hazard ratio in patients who received HCQ, unmeasured biases may exist, in addition to the limitations of a non-randomized, non-blinded observational trial.

### Studies suggesting no efficacy of HCQ for COVID-19 treatment

Several retrospective cohort analyses have failed to show efficacy of HCQ in virological clearance of COVID-19. Mallat et al. reported a delay in virological clearance in patients with COVID-19 treated with HCQ [67]. No improvement in lymphopenia or inflammatory markers was observed. This study was limited by small sample size (21 in HCQ and 13 in control arm) and exclusion of severe illness. A small case series of 11 patients with COVID-19 treated with HCQ + AZ revealed no virological clearance in eight patients 5–6 days after treatment [68]. The small sample size, lack of a control arm, and short follow-up were the limitations of this study. A prospective study measuring consecutive viral loads in 66 patients admitted with moderately severe COVID-19 did not find any difference in viral load clearance over time *in vivo* with use of HCQ compared to those not treated with HCQ [69]. Again, small sample size and variation in time for serial sampling were some limitations with this study. Similarly, another observational study evaluated the effect of HCQ on seroconversion in 43 patients with mild COVID-19 by PCR on Days 0.3 and 8 [70] and did not find any association of HCQ use with increase or decrease in viral RNA copy number. Small sample size, short follow up and exclusion of more severe disease were some of the limitations of this study. A retrospective observational study (*n* = 85) actually reported that use of HCQ + AZ was associated with decreased and delayed virological clearance, with median time to negative PCR being 23 days in HCQ + AZ arm and 19 days in control arm, and 77% patients in HCQ + AZ arm compared to 100% patients in control arm being PCR negative at Day 28 [71]. Again, small sample size in addition to a younger patient population, unequal use of other medications, and more symptomatic patients in HCQ + AZ arm were some limitations of this study.

Lack of efficacy of HCQ in clinical outcomes of COVID-19 has been observed in many retrospective cohort analyses. An observational study in patients with COVID-19, who required oxygen but not in an intensive care unit, from France did not observe any difference in clinical outcomes (survival or transfer to intensive care unit) in 84 patients who received HCQ and 89 patients who did not [72]. Furthermore, more patients had QTc prolongation in the HCQ arm than in the control arm. A retrospective observational study in 80 patients admitted to the ICU with severe COVID-19 did not find any difference in clinical outcomes (need for treatment escalation), ventilator free days, and mortality between the HCQ and control arm [73]. Another retrospective observational study (*n* = 84) in patients admitted with COVID-19 did not find any difference in risk of unfavourable clinical outcomes (death or transfer to ICU) in patients treated with HCQ and control arm [74]. Another observational analysis of 1,376 patients from New York determined that HCQ has no significant impact on intubation or death [75]. Although this analysis used a large sample size ensuring power, the confounding bias of unmeasured variables, such as older age and more comorbidities in the HCQ arm, must be considered. A retrospective cohort analysis of 4642 patients from France did not find any mortality benefit of HCQ ± AZ in patients hospitalized with COVID-19 [76]. Limitations of this study included more comorbidities in HCQ and HCQ + AZ arms, and lack of direct information on drug doses and study variables such as oxygen requirement. In another retrospective observational study of 2512 patients hospitalized with COVID-19, no difference in mortality rate was seen in patients prescribed HCQ ± AZ [77]. Significantly more patients who received HCQ were younger and less likely to be nursing home residents, although were more symptomatic. Further, there were variations in dosing, duration, and prescribing patterns of HCQ. Another large retrospective cohort analysis of inpatients with COVID-19 did not find any improvement in mortality or need for mechanical ventilation in those treated with HCQ ± AZ [78]. There were significant differences in baseline characteristics of the patients in either arm in this study. An investigation of 368 males in a Veterans Affairs hospital suggested no mortality benefit from HCQ ± AZ. The observers also suggested an increase in all-cause mortality with HCQ use [79]. Although a large sample was evaluated, the study population was all-male with more severe cases receiving HCQ ± AZ, possibly skewing the observed mortality increase with HCQ. A retrospective analysis of 1,438 New York hospital patients observed no significant difference in in-hospital mortality in patients receiving HCQ, AZ, and HCQ + AZ compared to no treatment [80]. As this was observational within a specific setting, the analysis of other hospital visits after discharge was limited. Only in-hospital deaths were measured, leaving the possibility for unmeasured deaths in another setting or hospital. A large multicenter retrospective analysis from the Netherlands (*n* = 1893) showed no difference in 21-day mortality in patients treated at hospitals that routinely used HCQ or CQ in patients admitted with COVID-19, compared to hospitals that did not [81]. Another study that suggested no survival benefit and increased risk of ventricular arrhythmias in patients with COVID-19 owing to HCQ or CQ was retracted owing to significant concerns with the accuracy of data acquisition and analysis [82,83]. Although retrospective analyses as mentioned above have the benefit of rapidly evaluating a hypothesis, several limitations exist including lack of randomization, risk of selection bias and confounding bias of unmeasured variables.

The first RCT evaluating the role of HCQ in COVID-19 by Chen et al. reported no benefit of HCQ in virological clearance, as 86·7% of patients in the HCQ arm and 90% in the conventional arm were nasopharyngeal swab PCR negative for SARS-CoV-2 by Day 7 [84]. No significant differences were noted in the resolution of fever or radiographic progression between groups. However, this study had several limitations, including the small sample size (15 patients in each arm), lack of intervention uniformity, and use of antiviral agents. Tang et al. also did not find any significant difference in clinical improvement time or PCR negativity in 70 patients treated with HCQ compared with 80 treated with the standard of care [85]. This study was open-label and antiviral treatments were used in both arms. Furthermore, the initial intention to treat protocol was not followed as several patients were switched to the other arm after the initial randomization, raising concerns of bias. Chen et al. reported no efficacy of HCQ in virological clearance in a multicenter open-label RCT (*n* = 33) with 81% patients in the HCQ arm and 75% patients in the standard of care arm being RT-PCR negative, with median time to negative PCR being 5 and 11 days respectively, none of these measures reaching statistical significance [86]. This study had small sample size, younger patients with only mild-moderate disease, and antivirals and antibacterials were used in the study. Another multicenter open-label RCT (*n* = 293) reported no difference in virological clearance at days 3 and 7 in patients treated with HCQ compared to patients in the control arm [87]. Unequal use of antivirals (more in HCQ arm), short follow up, lack of placebo masking, lack of blinding and overrepresentations of younger patients and healthcare workers (>80%) were some of the limitations of this study.

Use of HCQ ± AZ in 667 patients admitted with mild to moderate COVID-19 was evaluated by Cavalcanti et al. in a multicenter, randomized, non-blinded, open-label, three-group, controlled trial using a 7-level ordinal scale to evaluate the clinical status at day 15 [88]. Use of HCQ ± AZ was not associated with improvement in clinical status, need for mechanical ventilation, mortality rates, acute kidney injury and thromboembolic complications. QTc prolongation was observed in more patients treated with HCQ + AZ than those with HCQ alone or neither drug. This study was unblinded. Patients requiring oxygen 4 l/min or more were excluded from the trial. Protocol deviation was noted, and many patients had previously received HCQ and/or AZ 24 h prior to enrolment. Another single centre open label RCT evaluated the efficacy of HCQ in 500 patients admitted with mild COVID-19 [89]. No difference was noted in PCR negativity at Days 7 and/or 14, or the likelihood for disease progression between HCQ + standard of care arm and standard of care only arm. The study was unblinded and patients were mostly younger (35.96 ± 11.2 years), males (93.2), and all had mild infection with only 7.6% having comorbid conditions, limiting the ability of this trial to judge efficacy of HCQ in more severe cases.

The Randomized Evaluation of COVID-19 Therapy (RECOVERY) trial was established to evaluate the efficacy of several drugs, including HCQ, for COVID-19 [90]. On 5 June 2020, enrolment into the HCQ arm of the trial was stopped as preliminary data did not show any beneficial effect of HCQ in hospitalized patients with COVID-19. The preliminary results of this large (1561 HCQ arm, 3155 usual care arm) randomized, controlled clinical trial show no difference in the 28-day mortality rate between the HCQ (26·8%) and control (25%) arms [90]. Furthermore, patients in the HCQ arm had a lower probability of discharge, with a longer time to discharge, than patients in the control arm, with a higher probability of needing mechanical ventilation and death if mechanical ventilation was not used. No beneficial effect of HCQ on hospital stay duration was observed. As this trial included only hospitalized patients, with a mean duration of symptoms of 9 days and more than 75% needing some form of oxygen supplementation, the effects of HCQ earlier in the course of infection in patients with less severe illness could not be assessed. In another randomized, double-blind, placebo-controlled clinical trial evaluating the efficacy of HCQ among 423 outpatients with early COVID-19, the use of HCQ was not associated with reductions in the severity or duration of symptoms [91]. There was no statistically significant difference in hospitalizations or deaths between arms. This study was limited by the lack of cases with confirmed SARS-CoV-2 infection, with a PCR test performed on only 58% of the participants and 16% participants with a negative PCR test contributing to the data. The participants in this study were mostly “low-risk” with a median age of 40 years, 68% with no comorbidities, and only 3% being African-Americans, which limit the ability of this trial to inform the efficacy of HCQ for severe COVID-19 infection in higher-risk populations.

On 20 June 2020, The National Institutes of Health stopped the clinical trial to evaluate safety and efficacy of HCQ in patients hospitalized with COVID-19 after interim results did not show any benefit of HCQ compared to placebo [92]. On 4 July 2020, WHO discontinued the HCQ arm of the SOLIDARITY trial after reviewing the interim results, which showed no mortality benefit of HCQ in hospitalized patients with COVID-19 compared with the standard of care [93,94].

### Studies suggesting no prophylactic efficacy of HCQ for COVID-19

In a randomized, placebo-controlled, double-blinded trial of HCQ as a postexposure prophylactic initiated within 4 days after moderate- to high-risk exposure, HCQ was not associated with the prevention of illness compatible with COVID-19 [95]. This trial was limited by case definition (PCR confirmed or clinically compatible) and a lack of uniform PCR testing. The risk of asymptomatic infections could not be assessed owing to the lack of testing. Although patients in the HCQ arm experienced more adverse effects than those in the non-HCQ arm, most adverse effects were mild with no reports of arrhythmias, although an asymptomatic increase in QTc was not assessed.

The data available suggest that patients with rheumatic diseases (e.g. SLE) currently undergoing HCQ therapy remain at risk for COVID-19 and are not protected by HCQ use [96]. A group of 17 patients with SLE on long-term HCQ therapy who contracted COVID-19 progressed to severe disease despite baseline treatment with HCQ [97]. Data from the COVID-19 Global Rheumatology Alliance Global Registry show COVID-19 in 874 individuals with primary rheumatic disease, 27·4% of whom were on HCQ or CQ before COVID-19 diagnosis. There was no association between the use of HCQ and risk of hospitalization or serious infection in these patients, including those with SLE [98,99].

### Studies evaluating adverse effects of HCQ in COVID-19

The effect of HCQ with or without AZ on QTc prolongation has been investigated in several observational and case studies [10–15]. Significant QTc prolongation with HCQ with or without AZ was reported in 90% of patients in intensive care with COVID-19 (*n* = 40) by Bessière et al. [10]. Chorin et al. reported severe QTc prolongation (>500 ms) in 11% of patients (*n* = 84) treated with HCQ + AZ [11]. In anotherstudy, which included the previous 84 patients, severe QTc prolongation in 23% of patients (*n* = 251) was reported; eight patients discontinued therapy owing to severe QTc prolongation and one developed polymorphic ventricular tachycardia, suspected as TdP, needing cardioversion [12]. Ramireddy et al. reported critical QTc prolongation in 12% of patients (*n* = 490), with greater prolongation with the combination of HCQ and AZ than with either drug alone [13]. Saleh et al. also reported greater QTc prolongation with the combination of HCQ and AZ than with either drug alone (*n* = 210) [14]. Although these observational studies indicate critical QTc prolongation secondary to HCQ use in COVID-19, especially in combination with AZ, they have several limitations, including lack of a control arm and effects of confounding factors such as underlying comorbidities, disease severity, and other medications. A retrospective cohort analysis of 90 patients with COVID-19 treated with HCQ reported a significant increase in QTc interval, with a greater increase in those treated with AZ (53/90) [15]. Furthermore, this study reported one patient administered HCQ + AZ who developed TdP and other ventricular arrhythmias needing lidocaine. The limitations of this study included the lack of a control arm, short follow-up, small sample size, and effects of confounding factors such as underlying comorbidities, disease severity, and other medications. A transversal study evaluating effect of HCQ in ambulatory and admitted patients with COVID (*n* = 219) reported a significant but not clinically relevant increase in QTc from baseline of 416 ms to 423 ms [100] 48 h after treatment initiation with none of the participants showing an increase of more than 25% in QTc. The limitations of this study included the lack of a control arm, short follow-up, and exclusion of more severe disease. Finally, an analysis from 3 RCTs evaluating HCQ as pre-exposure prophylaxis, post-exposure prophylaxis, and early treatment in COVID-19 reported safety data on HCQ in this population (*n* = 2795) [101]. Patients treated with HCQ experienced more adverse effects, mostly gastrointestinal and mild. Only one patient in the HCQ arm developed supraventricular tachycardia and no cases of sudden cardiac death or ventricular arrhythmias were noted, although specific effects of HCQ on QTc were not evaluated. Limitations of this analysis included inclusion of only outpatient and mostly younger, healthcare worker participants with less underlying comorbidities, thus excluding those with more severe disease and more comorbidities.

### Summary of available literature

This review of the available literature suggests a scarcity of well-conducted clinical trials on HCQ for COVID-19 (Table 2). While the initial observational data suggested possible efficacy of HCQ in COVID-19, recent clinical trial data has largely been unable to reproduce these results. However, several methodological drawbacks have been noted in the available literature so far. Further, the currently available data is insufficient to definitively confirm or rule out cardiotoxicity from HCQ when used in COVID-19. However, this concern retains significance as most critical patients infected with SARS-CoV-2 have underlying cardiac comorbidities [9]. Owing to the paucity of research in COVID-19, recommendations for or against therapy cannot be suggested in earnest [102]. It is appropriate to remain mindful of potential cardiovascular risk when prescribing HCQ, especially to those with comorbidities.

**Table 2.** Summary of COVID-19 studies involving hydroxychloroquine.

Date	Authors	Study type	N	Study results (HCQ associated with:)	Study limitations
March 2020	Gautret et al. [57]	Cohort	36	Improved viral clearance	Obs, SS, no randomization, unblinded, LTFU all in HCQ group
March 2020	Chen J. et al. [84]	RCT	30	No improvement in viral clearance, mortality	SS, no intervention unifromity, antivirals used
March 2020	Chen Z. et al.[59]	RCT	62	Improved time to clinical recovery	SS, severe cases excluded, inadequate primary end points
March 2020	Molina et al. [68]	Case series	11	No improvement in viral clearance	Obs, SS, no control arm, short follow up
April 2020	Gautret et al. [58]	Case series	80	Improved clinical course and viral clearance	Obs, SS, no control arm, changes to viral PCR threshold
April 2020	Mathian et al.[97]	Case series	17	No impact on clinical course in patients with SLE	Obs, SS, obs., Rheumatic population
April 2020	Chorin et al. [11]	Case series	84	QTc prolongation	SS, no control arm, effect of other medications, severity of illness, CM
April 2020	Saleh et al. [14]	Case series	201	QTc prolongation	Obs, no control arm
April 2020	Magagnoli et al. [79]	Cohort	368	No improvement in mortality, intubation. Increased all-cause mortality	Obs, all male, more severe cases received HCQ +/- AZ
April 2020	Ramireddy et al. [13]	Case series	98	QTc prolongation	Obs, no control arm
May 2020	Ahmad et al. [61]	Case series	54	Improved clinical recovery	Obs, no control arm, clinically worsened patients not included in final analysis
May 2020	Yu et al. [63]	Cohort	568	Improved mortality and decreased IL-6 levels in critically ill	Obs, use of antivirals. Unequal use of other medications
May 2020	Bessière et al. [10]	Case series	40	QTc prolongation	Obs, Early discontinuation, use of other cardiotoxic drugs, ICU patients, CM
May 2020	Chorin et al. [12]	Case series	251	QTc prolongation	Obs, No control arm, effect of other medications, severity of illness, CM
May 2020	Mercuro et al. [15]	Cohort	90	QTc prolongation	Obs, SS, no control arm
May 2020	Mallat et al. [67]	Cohort	34	Dealy in viral clearance, no improvement in lab-markers	Obs, SS, exclusion of severe illness
May 2020	Gianfrancesco et al. [98]	Case series	600	No reduction in hospitalization in patients with rheumatic diseases	Obs, Retrospective analysis, focus beyond HCQ in rheumatic populations
May 2020	Novales et al. [64]	Cohort	166	Improved mortality	Obs, SS, use of other medications, potential baseline confounders
May 2020	Million et al. [62]	Case series	1061	Improved viral clearance, mortality, clinical outcomes	Obs, No control arm, therapy unsupervised, incomplete data
May 2020	Geleris et al. [75]	Cohort	1376	No improvement in mortality, intubation	Obs, HCQ arm with older age, more comorbidities
May 2020	Rosenberg et al. [80]	Cohort	1438	No improvement in clinical outcomes or mortality in inpatients	Obs, only in-hospital deaths measured
May 2020	Tang et al. [85]	RCT	150	No improvement in viral clearance, time to clinical recovery	Open label, antivirals in both arms, patients switched arms
May 2020	Mahévas et al. [72]	Cohort	181	No improvement in ICU transfer, mortality	Obs, no randomization, potential baseline confounders
May 2020	Singh et al. [78]	Cohort	3372	No improvement in mortality and need for mechanical ventillation	Obs, potential baseline confounders
May 2020	Ip et al. [77]	Cohort	2512	No improvement in mortality	Obs, no randomization, variation in HCQ prescribing patterns
June 2020	Ferreira et al. [65]	Cohort	360304	Less odds of PCR positivity	Obs, missing information on comorbidities, compliance
June 2020	Paccoud et al. [74]	Cohort	84	No improvement in clinical outcomes	Obs, SS, no randomization,
June 2020	Faico-Filho et al. [69]	Cohort	66	No improvement in viral clearance	Obs, SS, variable time for serial sampling
June 2020	Boulware et al. [95]	RCT	821	No efficacy as post-exposure prophylaxis	Case definiation limitation, lack of uniform PCR testing
June 2020	Sbidian et al. [76]	Cohort	4642	No improvement in mortality	Obs, lack of direct information on study variables
June 2020	Chen L et al. [60]	RCT	48	Improved time to clinical recovery	SS, unblinded, terminated early, underpowered
June 2020	Arshad et al. [66]	Cohort	2541	Improved mortality	Obs, effect of other medications
July 2020	Lecronier et al. [73]	Cohort	80	No improvement in clinical outcomes or mortality in ICU patients	Obs, SS, unblinded, more severe illness
July 2020	Horby et al. [90]	RCT	4716	No improvement in mortality, more death and ventillation in non-ventillated patients	More severe illness, therapy initiated after prolonged illness
July 2020	Cavalcanti et al. [88]	RCT	667	No improvement in clinical status	Unblinded, protocol deviation
July 2020	Mitja et al. [87]	RCT	293	No improvement in viral clearance	Unblinded, unequal use of other medications, younger patients
July 2020	Chen CP et al. [86]	RCT	32	No improvement in viral clearance	SS, exclusion of severe illness, antivirals used
July 2020	Skipper et al. [78]	RCT	432	No improvement in symptom duration or severity in outpatients	Lack of uniform PCR testing, healthier and lower-risk participants
July 2020	Komissarov et al. [70]	Cohort	43	No improvement in viral clearance	Obs, SS, short follow up, exclusion of severe illness
August 2020	Peters et al. [81]	Cohort	1893	No improvement in mortality	Obs, variability in standard of care, unequal use of other medications
August 2020	Saleemi et al. [71]	Cohort	85	Delayed and decreased chances of virological clearance	Obs, SS, unequal use of other medications, potential baseline confounders
August 2020	Lofgren et al. [101]	RCT	2719	More GI adverse effects (mild), no ventricular arrythmias or sudden cardiac death	Younger healthier participants, exclusion of severe illness
August 2020	Jaimez et al. [100]	Case series	219	Significant but not clinically relevant QTc prolongation	Obs., No control arm, exclusion of severe illness
August 2020	Kamran et al. [89]	RCT	500	No improvement in disease progression and PCR conversion	Unblinded, younger, healthier, male patients with mild illness.

SS: small sample; obs.: observational limitations; CM: co-morbidities; LTFU: Lost to follow up; N-PCR: Nasopharyngeal Polymerase Chain Reaction; Green: Showing efficacy of HCQ; Red: Showing no efficacy of HCQ; Yellow: Studying adverse effects of HCQ.

As early literature suggested the efficacy of HCQ against COVID-19, several organizations [103,104] supported the cautionary use of this medication. For example, the Food and Drug Administration (FDA) approved HCQ for emergency use [103]. Although these early studies were crucial steps, recent trials suggest that the purported benefits of this medication may not outweigh the potentially life-threatening adverse effects. Owing to the evolving literature, several agencies have now advised against HCQ administration. The Centre for Disease Control, FDA, European Medical Agency, American College of Physicians, Infectious Disease Society of America, and National Institutes of Health have publicly stated the need for caution when prescribing HCQ for COVID-19 outside hospital and clinical trial settings [105–110].

## Conclusion

HCQ, initially an antimalarial agent, is used as an immunomodulatory agent for managing several autoimmune diseases, for which its efficacy is linked to inhibiting lysosomal antigen processing, MHC-II antigen presentation, and TLR functions. It is generally well-tolerated although severe life-threatening adverse effects have been reported. HCQ gained popularity as a potential therapy for COVID-19, owing to *in vitro* data suggesting its antiviral activities by interfering with lysosomal functions. However, data on its efficacy and safety in COVID-19 are still insufficient, with several methodological difficulties and small sample sizes. Recent clinical trials suggest no role of HCQ in COVID-19 treatment or prevention, and there are unanswered questions about its cardiac safety in patients with COVID-19. Until further randomized controlled trials eliciting the efficacy and safety are available, HCQ use in COVID-19 should be discouraged outside clinical trials under strict medical supervision. Although rapid publication of small trials and observational analyses are necessary during a global pandemic, well-performed clinical trials with better methodology will best present reliable and valid data moving forward. Further low-powered investigation will only continue to cloud the overall information on this topic. Although it may not be possible to perform flawless clinical trials, researchers should plan future trials by assessing the limitations of published studies to achieve high-quality research with minimal bias and few methodological errors.

## Data Availability

The authors confirm that the data supporting the findings of this study are available within the article.
